# In it Together: Sense of Community and Psychologic Distress During COVID-19

**DOI:** 10.21203/rs.3.rs-2838471/v1

**Published:** 2023-05-03

**Authors:** Kimberly Wu, Erica Doe, Gabriela Roude, Jasmine Wallace, Samantha Francois, Lisa Richardson, Katherine Theall

## Abstract

We assessed the relationship between differences in indicators of social capital before and during the COVID-19 pandemic, and their association with self-reported measures of psychological distress. The data was analyzed from an existing cluster randomized control trial (the *Healthy Neighborhoods Project* ) with 244 participants from New Orleans, Louisiana. Differences in self-reported scores between baseline (January 2019-March 2020) and participant’s second survey (March 20, 2020, and onwards) were calculated. Logistic regression was employed to examine the association between social capital indicators and measures of psychological distress adjusting for key covariates and controlling for residential clustering effects. Participants who reported higher than average scores for social capital indicators were significantly less likely to report increases in psychosocial distress between pre and during the COVID-19 pandemic. Those who reported higher-than-average sense of community were approximately 1.2 times less likely than those who reported lower than average sense of community scores to experience increases in psychological distress before and during the global pandemic (OR = 0.79; 95% CI = 0.70,0.88, p ≤ 0.001), even after controlling for key covariates. Findings highlight the potentially important role that community social capital and related factors may play in the health of underrepresented populations during times of major stress. Specifically, the results suggest an important role of cognitive social capital and perceptions of community membership, belonging, and influence in buffering changes of mental health distress experienced during the initial period of the COVID-19 pandemic among a population that is majority Black and female.

### INTRODUCTION

With the official declaration of the global pandemic in March 2020, there has been extensive attention given to tracking its impact on public health in the United States. As highlighted by many engaging in the discourse around health equity, the compounded syndemic of structural racism amplifies the effects of underlying systemic barriers and macro-level racism, adding additional strains to the mental health and well-being of communities of color.[1] Increasing psychological distress has been shown to be associated with increased risk of poor health across most populations, with evidence of higher rates of chronic disease, asthma, cancer, and diabetes.[2] There is growing evidence that individuals with pre-existing physical conditions of hypertension, chronic kidney disease, diabetes, cardiovascular disease, cerebrovascular disease, and chronic obstructive pulmonary disease are at a higher risk for hospitalization and early death when diagnosed with COVID-19.[3] Furthermore, there are mixed findings from research exploring the association between pre-existing mental health diagnosis with increased risk for COVID-19 related hospitalization and mortality.[3]

Although we may not understand the mechanisms through which mental health may affect individuals' risk for COVID-19 related health outcomes, we have ample evidence that Black, Indigenous and communities of color have experienced higher rates of COVID-related mortality.[4,5] Across the country, there is a continuous pattern that Black, Latinx, and individuals of marginalized identities have been adversely affected by the pandemic. A survey from the Commonwealth Fund found that Latinx and Black adults reported experiencing economic hardships almost 2 to 3 times more than White adults at early stages of the pandemic.[6] In national surveys, more people of color reported experiences of anxiety and depression.[7] Given the deep and lasting history of systemic racism in the US, Black and Brown communities continue to be disproportionately affected by the compounded stressors exacerbated by instabilities and uncertainties associated with the pandemic's everchanging progress.[4,8,9]

Addressing how social determinants of health are affecting families during the pandemic can be difficult, but there is opportunity to investigate how resiliency may be strengthened while navigating moments of uncertainty. Research into global crises reveals possibilities for buffering psychological distress through increasing meaningful social connections and fostering a sense of community. Findings from studies on mental health resilience among communities of color highlight mechanisms that may be particularly salient. A recent study on the trajectories of psychological resilience and mental health distress throughout a period from March 10 to August 4, 2020, highlights a protective relationship among adults reporting high levels of resilience (as measured by the Brief Resilience Scale) with lower odds of mental distress.[10] Additionally, the authors reported populations within their sample including, males, Black adults, those over 50 years old, and adults with graduate degrees had higher odds of reporting higher resilience, as compared to individuals living below the poverty line.[10]

Connectedness and resilience can be indicators of social capital, which as an overarching concept has been defined in many ways. Cohen and Prusak[11] offer this definition of social capital as "active connections among people: the trust, mutual understanding, and shared values and behaviors that bind the members of human networks and communities and make cooperative action possible". This conceptualization of social capital highlights the agency that relationships and connections can inspire. Furthermore, focusing on indicators of social capital within neighborhood spaces present opportunities for understanding how relationships among neighbors and within local networks can have an effect on mental health and psychological distress. Aspects of social capital have been affected by changes brought on by the pandemic, including major shifts to patterns of socialization and employment as a result of stay-at-home orders, school shutdowns, and upholding guidance related to physical distancing. While there is evidence of redefined patterns to socializing, including the unique role of social media and technology, there are still barriers faced by communities in neighborhoods with limited access to public spaces, poor conditions of public spaces, or concerns of crime and safety.[12–14]

Given the impact of the challenges presented by the pandemic on mental health and the important role that neighborhood conditions may play in buffering or exacerbating pandemic related stress, this study aims to explore how dimensions of social capital (operationalized as sense of community and neighborhood collective efficacy) are associated with changes in self-reported psychological distress and perceived stress among a sample of primarily Black residents living in low-income neighborhoods in New Orleans, Louisiana–an area experiencing great inequities in rates of COVID-19 infection, morbidity, and mortality[15]. In this study, we examine how self-reported indicators of social capital among residents in

New Orleans affect changes in self-reported psychological distress and perceived stress following the first several months into the COVID-19 pandemic. We hypothesized that residents with higher measures of social capital, operationalized as indicators for greater perceived sense of community and collective efficacy, would experience less self-reported distress and perceived stress before and during the COVID-19 pandemic time periods.

## METHODS

The study draws on secondary survey data from an existing cluster randomized control trial funded by the National Institute of Health (NIH, R01HD095609) with additional funding support from the Robert Wood Johnson Foundation's Evidence for Action Program. The parent study, referred to as the *Healthy Neighborhoods Project (HNP)*, is taking place across 23 neighborhoods in New Orleans, and is aimed at testing the impact of neighborhood beautification on violence prevention and overall health. The parent study includes a longitudinal cohort portion that follows the experiences of neighborhood residents throughout the 5-year project. The current study sample is a sub-sample of 244 respondents with both baseline data collected before the COVID-19 pandemic (January 2019-March 2020), and Wave 2 data collected following the stay-at-home orders from city officials beginning March 20, 2020, and onwards.[16] All methods in the study are in accordance with relevant guidelines and regulations.

Data collection. Trained interviewers collected survey data utilizing RedCap™ software in person (5%, n = 12) and over the phone (95%, n = 232). Participants with different modes of data collection did not differ in any way according to sociodemographic factors, exposures, or outcomes of interest in the present study. Both baseline and Wave 2 surveys took approximately 45 minutes to complete, and respondents provided consent to participate before enrollment. The Tulane University Institutional Review Board approved the study.

Measures. **Dependent variables**. To capture differences in self-reported mental health distress and stress, the Kessler 6 (K6) Psychological Distress Scale and Perceived Stress Scales (PSS) were included in the parent study. The K6 scale measures the frequency of "non-specific psychological distress" with six items, each ranging from zero for "none of the time" to four for "all of the time". Questions focus on negative feelings/emotions and related ability to carry out normal activities and care-seeking.[17] The items are reduced into a summary score, with higher scores indicating greater psychological distress. Scores of 5 and over indicate moderate psychological distress and 13 and over indicate serious psychological distress.[17] The index demonstrated internal reliability in the parent sample (Cronbach's alpha = 0.89).

The PSS was measured using four items where each item was scored ranging from zero for "never" to four for "very often". Positive items were reverse coded and then a total score was obtained by aggregating across all 4 items.[18] The scale also demonstrated internal reliability in the parent sample (Cronbach's alpha = 0.71). To examine change in self-reported mental health, difference scores for K6 and PSS scores were calculated between each participant's baseline (pre-pandemic) and Wave 2 (during pandemic) responses. An increase in change scores for either measure indicated an increase in self-reported mental health distress or perceived stress over time and was coded as one to indicate a decrease or zero to indicate no change.

**Independent variables.** Social capital was assessed with two separate indicators—*Sense of Community* and *Collective Efficacy*. Sense of community was measured using the sense of community index (SCI), a validated tool that measures perceptions of connection and membership to a group or community. The SCI includes 12 items, scored on a "mostly true/mostly false" scale for each item, resulting in total scores ranging from zero to twelve. Lower scores indicate a lower perception of a sense of community.[19] The SCI also included two subscales to measure emotional connection to community as well as sense of belonging to community or membership. Each subscale was made up of six questions.[19] The SCI scale was designed to include an overall score and subscales scores that capture more specific constructs for sense of membership, sense of influence, reinforcement of needs, or shared emotional connection.[19] Scale items included responding to statements such as, "I can recognize most of the people who live in my neighborhood", "I care about what my neighbors think of my actions", "I have influence over what this neighborhood is like", and "If there is a problem in this neighborhood people who live here can get it solved". Within our sample, the SCI demonstrated strong reliability (Cronbach's alpha = 0.85).

In addition to incorporating the SCI, neighborhood collective efficacy was assessed with survey items taken from measures developed by Sampson and colleagues[20] which included 8 items scored on a 5-point scale ranging from 1 ("strongly disagree") to 5 ("strongly agree"), for total scores ranging from 8 to 40. To measure collective efficacy, participants responded to statements such as, "You can count on adults in this neighborhood to watch out that children are safe and don't get into trouble", "The police protection in my neighborhood is adequate", "I can trust the government in New Orleans to do what is right for my neighborhood", and "If there is a problem around here, the neighbors get together to deal with it". Lower scores indicated a lower sense of perceived neighborhood collective efficacy. The measure demonstrated reliability in our sample (Cronbach's alpha = 0.85).

**Covariates.** The covariates included in the study are related to the sociodemographic backgrounds of the participants. These variables included age, sex, relationship status, education, employment status, and participant attitudes about COVID-19 prevention. *Age* was measured continuously and ranged from 20 to 80 years. The responses were recoded into 9-year categories, "20–29", "30–39", "40–49", "50–59", "60–69", and "over 70". *Sex* was measured as a binary variable where 1 indicates "female", and 0 indicates "male". *Relationship status* was measured in the categories of "married", "living with a partner", "divorce/separated", "widowed", "single/never married", and "multiple". A response of "multiple" indicates that the respondent may experience multiple relationship statuses at once. The categories of "living with a partner" were combined with "married" and, "never married" with "multiple" and "other", to address the small percentage of responses (less than 5%). Education attainment included categories of "less than high school education", "high school/GED", "some college", "college", and "graduate" levels. *Employment status* was recoded so that each category had more than 5% of the sample size for analysis. The categories are "full time", "part-time", "unemployed", "unable to work because of a disability", and "other".

Other included individuals who identified as retired, full-time homemakers, and individuals in school or a training program. *Attitude around COVID-19 prevention* was measured as participant's response to the statement "If I don't take preventative action, then I am likely to get COVID-19." Participants responses were recoded into three categories- "probably true", "probably false", and "not sure/no opinion".

## STATISTICAL ANALYSIS

**Data Analysis.** Descriptive, bivariate, and multivariate analyses were conducted, including chi-square and t-test for categorical and continuous bivariate analyses, respectively. Logistic regression was employed to examine the association between social capital indicators and measures of psychological distress (K6 and PSS), adjusting for key covariates. Regression models were analyzed separately for each measure of social capital and mental health outcome—increases in psychological distress or perceived stress—before March 2020 and during the COVID-19 pandemic from March 2020 vs. no change or decrease. All analyses were performed with Stata/BE 17.0.

### RESULTS

Slightly more than half of the total sample population reported experiencing an increase in psychological distress between baseline and Wave 2 data collection. Table 1 presents the sociodemographic background data of individuals involved in the study, stratified by changes in psychological distress from the pre-COVID onset and during the pandemic. The average age of participants was 52 years. About 70% of the sample identified as female and 30% as male. A majority self-identified as non-Hispanic Black/African American (77%), roughly 15% identified as non-Hispanic White, with 4% identifying as non-Hispanic other, and the remaining 4% identifying as other (ethnically Hispanic). Nearly 40% of study participants identified as single, a little over 25% identified as married or living with a partner, 10% identified as widowed, 11% identified as divorced or separated, and the remaining 14% identified being in other types of relationship statuses. Almost 39% of the participants identified having full-time employment. Among respondents who reported an increase in psychological distress, 10% reported experiencing unemployment, as compared to 5% of respondents who reported a decrease or no change in psychological distress before and during the pandemic.

For the study sample, the average perceived neighborhood collective efficacy score was 28 (range from 8–40), with scores slightly lower for participants who experienced an increase in distress from before-during the pandemic ($p < 0.05$). Furthermore, the average sense of community score was a little over 9, with those who reported experiencing an increase in distress scoring slightly lower scores (although the association was not significant).

Table 2 presents the results from crude and adjusted logistic regression models for each marker of social capital (sense of community index and collective efficacy). From the crude models, respondents reporting a greater sense of community were significantly less likely to experience an increase in psychosocial outcomes over time, for both measures of psychological distress (K6) (OR = 0.51, 95% CI = 0.29, 0.91, $p \leq$

0.05) and perceived stress (PSS) (OR = 0.49, 95% CI = 0.28, 0.87, $p \leq 0.05$). Crudely, respondents who reported higher levels of neighborhood collective efficacy also reported lower odds of increased psychological distress over time for both K6 (OR = 0.40, 95% CI = 0.23, 0.71, $p \leq 0.05$) and PSS (OR = 0.69, 95% CI = 0.40, 1.19) measures.

When adjusting for the effects of age, sex, relationship status, education level, employment, attitude around COVID-19 prevention, and cluster-level design, both measures of social capital continued to exhibit significant and protective associations with changes in psychosocial outcomes. Study participants who reported a higher-than-average sense of community were approximately 1.2 time less likely than those who reported lower than average sense of community scores to experience increases in psychological distress before and during the global pandemic (OR = 0.79; 95% CI = 0.70,0.88, $p \leq 0.001$) when controlling for covariates. Furthermore, the same participants were also slightly less likely than those who reported lower than average sense of community scores to experience increased perceived stress differences (OR = 0.84; 95% CI = 0.75, 0.95, $p \leq 0.01$).

From the models, higher neighborhood collective efficacy scores were associated with significantly less increases in psychosocial outcomes between pre-March 2020 with COVID-19 pandemic timelines, although the effects were smaller. Participants who reported higher-than-average neighborhood collective efficacy scores were 10% less likely than those who reported lower-than-average scores to experience increases in distress over time (OR = 0.90, 95% CI = 0.85, 0.95, $p \leq 0.001$) when controlling for relevant variables. In addition, participants with higher-than-average neighborhood collective efficacy scores were 8% less likely than those who reported lower-than-average-scores to experience increased perceived stress scores over time (OR = 0.92, 95% CI = 0.87, 0.97, $p \leq 0.01$).

Table 1
Individual and neighborhood socio-demographic characteristics of study participants stratified by psychological distress before and during COVID 191

| | Increase in psychological distress<br><br>(N = 116) | No change or decrease in before psychological distress<br><br>(N = 128) | Total<br><br>(N = 244) |
|---|---|---|---|
| | **Mean (range) / %** | **Mean (range) / %** | **Mean (range) / %** |
| **Average age (years)** | 50.90 (22–83) | 53.38 (22–94) | 52.20 (22–94) |
| **Sex*** | | | |
| Female | 69.83% | 69.53% | 69.67% |
| Male | 30.47% | 30.17% | 30.33% |
| **Self-reported racial identity** | | | |
| Non-Hispanic Black | 77.59% | 77.34% | 77.46% |
| Non-Hispanic White | 12.93% | 16.41% | 14.75% |
| Non-Hispanic Other | 5.17% | 3.13% | 4.10% |
| Other (Hispanic) | 4.31% | 3.13% | 3.69% |
| **Relationship status** | | | |
| Married/living with a partner | 26.72% | 24.22% | 25.41% |
| Divorced/Separated | 9.48% | 12.50% | 11.07% |
| Widowed | 10.34% | 10.16% | 10.25% |
| Single | 38.79% | 39.84% | 39.34% |
| Other (Multiple/Never married) | 14.66% | 13.93% | 13.93% |
| **Employment status** | | | |
| Full-time | 37.93% | 39.06% | 38.52% |
| Part-time | 12.93% | 16.41% | 14.75% |
| Unemployed | 9.48% | 4.69% | 6.97% |
| Unable to work due to disability | 14.66% | 17.19% | 15.98% |
| Other | 25.00% | 22.66% | 23.77% |

| | Increase in psychological distress<br><br>(N = 116) | No change or decrease in before psychological distress<br><br>(N = 128) | Total<br><br>(N = 244) |
|---|---|---|---|
| | **Mean (range) / %** | **Mean (range) / %** | **Mean (range) / %** |
| **Education** | | | |
| Less than high school | 5.31% | 13.71% | 9.70% |
| High school graduate | 32.74% | 26.61% | 29.54% |
| Some college | 36.28% | 24.19% | 29.96% |
| 4-year college | 17.70% | 22.58% | 20.25% |
| Graduate or professional school | 7.96% | 12.90% | 10.55% |
| **Average perceived neighborhood collective efficacy*[2]** | 26.10 | 28.94 | 27.57 |
| **Average sense of community index score[3]** | 8.96 | 10.36 | 9.69 |

[1] *Psychological distress measured by Kessler Psychological Distress Scale with scores ranging from 10–50 where higher scores indicate greater distress.*

[2] *Percevied neighborhood collective efficacy eight items measured on a five-point scale where higher scores indicate higher collective efficacy.*

[3] *Sense of community index measures the aggregate score of true/false (true = 1, false = 0) responses for 12 items that capture subscale responses of sense of membership, integration, influence, and shared emotional connection with scores ranging from 0–12.*

[4] *Neighborhood attachment score includes 7 items measured on a four-point scale where a higher score indicates higher neighborhood attachment.*

**Statistically significant difference at $p \leq 0.05$.*

*Notes. Mean / % of any variable based on < 10% missing.*

Table 2

Impact of Social Capital on Psychologic Distress and Perceived Stress Differences Before and During COVID-19: Results of Crude and Adjusted Logistic Models

| | Crude Models | | Adjusted Models[1] | |
|---|---|---|---|---|
| | Odds Ratio | 95%CI | Odds Ratio | 95% CI |
| **Psychological Distress Differences Outcome (K6)** | | | | |
| Model 1: Sense of community | 0.51* | (0.2911, 0.9054) | 0.79§ | (0.7038, 0.8834) |
| Model 2: Neighborhood Collective Efficacy | 0.40* | (0.2292, 0.7099) | 0.90§ | (0.8523, 0.9543) |
| **Perceived Stress Differences Outcome (PSS)** | | | | |
| Model 3: Sense of community | 0.49* | (0.2824, 0.8652) | 0.84** | (0.7458, 0.9488) |
| Model 4: Neighborhood Collective Efficacy | 0.69 | (0.4011, 1.1867) | 0.92** | (0.8739, 0.9747) |

[1] *Controlling for age categories, sex, relationship status, education, employment, attitudes around COVID-19 prevention, and cluster-level effects.*

**Note**

*Modeling difference score for both K6 and perceived stress difference. A greater difference score indicates an increase in psychological distress and perceived stress following the start of the COVID pandemic*

## *= statistical significance at $p < 0.05$

## **=statistical significance at $p \leq 0.01$

## §= statistical significance at $p \leq 0.001$

### DISCUSSION

This study contributes to the growing literature on the role that social capital may play in buffering pandemic stress, with both indicators of social capital reflecting modest protective effects from increased psychological distress and perceived stress before and during the COVID-19 pandemic. Our models showed that residents who indicated higher measures of sense of community and collective efficacy were also less likely to report increases in psychological distress and perceived stress at the moment of our study and its timing during the early stages of the pandemic.

Although the general consensus on the relationship between social capital and its effects on mental health is mixed, these results corroborate findings on the beneficial role of social capital for mental health, where higher levels of social capital has been found to be inversely related to mental illness diagnosis in community and individual contexts.[21] Studies linking measures of social capital and specifically the Kessler 6 measures of psychological distress have led to results that support our findings. Relevant studies have reported findings in which lower levels of community social capital were associated with psychological distress.[22,23] The benefits of social relationships on a more general level are connected with facilitating positive physiological and cognitive responses that minimize stress or partaking in risky behaviors.[24] Furthermore, there is some evidence that lower perceptions of social support and smaller networks of social connections are associated with depressive symptoms.[25]

Our findings also add to the growing literature that supports a key role of social capital on outcomes related to the COVID-19 pandemic and recovery from crisis situations.[26] Psychological responses to navigating “home confinement” and quarantine during the early stages of the pandemic have been documented, including negative emotional outcomes and stress regarding managing the burdens and logistics of securing day-to-day supplies, finances, and other familial needs.[27] In response, fostering social capital through strengthening community and familial connections is recognized to promote more trust and sharing of information to inspire preventative action and preparation beneficial to navigating changes during this time.[26,28] In addition, social capital is theoretically linked to promoting civic norms outside of formal institutions, and may serve to unite residents to work towards shared goals and fostering a greater sense of the value of contributing towards collective action.[28]

Finally, this study adds to the existing literature by including experiences of racial and ethnic minoritized groups.[25] From a similar study in the UK, researchers identified that social capital and psychological distress were distributed differently across Black and ethnic minorities, raising the importance of considering diverse experiences across gender and racial identities.[25] While this study does not analyze outcomes by racial and ethnic subgroups, it focuses on a sample of predominantly Black female residents living in New Orleans, Louisiana. Specific to New Orleans, COVID-related mandates, and phases of the city’s reopening since March 2020 have had varying impacts on the economic, social, and health equity-related outcomes.[29] Black men and women representing a majority of the city’s population were disproportionately represented among the ranks of essential and frontline workers in New Orleans, and Black women have been further affected by job losses related to COVID-19 and lower wages for their labor.[30] The strains and burdens experienced by the Black communities in New Orleans were further amplified by inequities in rates of COVID-19 infection, morbidity, and mortality.[15] Additional research into the nuances of social networks and specific mechanisms of buffering for psychological distress are relevant to better understand the health realities and priorities for Black residents in New Orleans, Louisiana, and the Gulf South at large.[31]

Our findings reflect a protective element of social capital among respondents who reported higher than average sense of community and collective efficacy scores. One area of critique related to social capital raises the need for introducing greater complexity and nuance when teasing out the underlying mechanisms and theories that motivate the formation of social connections and networks.[32] We were not necessarily able to address this critique in our study directly, but it provides us with direction for future projects through the incorporation of ethnic identity measures and validated scales to assess the potential moderating effects of perceived racial or ethnic identities and relationships to social capital and mental health.

One aspect that may bridge individual social capital (individual perceptions of the community) to community and institutional social capital within the Black community in New Orleans may be connected to strong historical and contemporary practices of grassroots mutual aid efforts in New Orleans.[33] Mutual aid is a form of civic participation, where people take on the responsibilities for direct care for one another beyond symbolic acts and into creating new relational patterns and structures to meet folks' immediate needs.[34] Mutual aid has garnered more national attention during COVID-19 under the work of local institutions and groups led by communities of color, many of whom are directly affected by structural inequities, and have continued to sustain their efforts to provide resources to community members.[34] Within New Orleans, and Louisiana more broadly, mutual aid societies continue to flourish to address the needs of historically marginalized groups. Raising visibility of the organized capacity of marginalized groups to exercise agency and materially provide for each other's needs is an important narrative to counter claims that groups of color are "dependent" on institutions and labeled "resource poor" in a way that perpetuates unbalanced stereotypes and perceptions of communities of color.[33]

Despite our findings, there are several limitations to this study that must be raised. Primarily, the findings were drawn from a small and select sample of residents and are based on self-reported data which is subject to recall and social desirability biases. Findings from the study cannot be generalizable to all residents in New Orleans but serve as a helpful reminder to invest in more holistic conceptions of mental health that draw connections between individuals and community life. While the sample size was small, it is representative of many low-income neighborhoods in the city. Finally, there are limitations to our indicators of social capital, as they do not capture the full conceptualization of the construct. An ongoing consideration related to operationalizing social capital includes clarifying the type and level of focus of the measures utilized. Generally, measures of sense of community and collective efficacy fall within cognitive forms of social capital that capture community-level characteristics as opposed to individual-level traits.[35] As demonstrated from our findings, the sense of community index was found to be a stronger buffer of psychosocial change scores as compared to the measure for neighborhood collective efficacy among the sample population. Neighborhood collective efficacy may differ from sense of community by capturing dimensions of neighborhood capacity that extend beyond perceptions of social connection into the realm of engagement in shared action.

## CONCLUSION

Findings from this study highlight the value of conducting additional research into the relationship of measures of social capital and particular factors most relevant to underrepresented populations. Specifically, the results suggest an important role of cognitive social capital and perceptions of community membership, belonging, and influence in buffering changes of mental health distress experienced during the initial period of the COVID-19 pandemic among a population that is majority Black and female across 23 neighborhoods of New Orleans, Louisiana. Furthermore, incorporating social capital and its relevance for highlighting existing and long-standing grassroots and community structures are useful for identifying norms and practices that address local needs and may play a role in promoting resilience.[32] Additional research, partnership, and advocacy into the importance of interdisciplinary national efforts to support local mechanisms for fostering social connection and belonging, such as through mutual aid groups can also support mental health, and is an avenue that requires added attention.

## Declarations

#### DATA AVAILABILITY

The repository for the related dataset can be found at the following Box link: https://tulane.box.com/s/ox7h2i4isp8ri7euula6b969blux6585.

## References

1. Shim RS, Starks SM. COVID-19, Structural Racism, and Mental Health Inequities: Policy Implications for an Emerging Syndemic. *https://doi.org/101176/appi.ps202000725*. 2021;72(10):1193-1198. doi:10.1176/APPI.PS.202000725
2. Barry V, Stout ME, Lynch ME, et al. The effect of psychological distress on health outcomes: A systematic review and meta-analysis of prospective studies. *J Health Psychol*. 2020;25(2):227-239. doi:10.1177/1359105319842931
3. Egede J, Campbell JA, Walker RJ, Garacci E, Dawson AZ, Egede LE. Relationship between physical and mental health comorbidities and COVID-19 positivity, hospitalization, and mortality. *J Affect Disord*. 2021;283:94-100. doi:10.1016/J.JAD.2021.01.048
4. COVID Tracking Project. The COVID Racial Data Tracker | The COVID Tracking Project. *The Atlantic*. Published online 2021.
5. Andraska EA, Alabi O, Dorsey C, et al. Health care disparities during the COVID-19 pandemic. *Semin Vasc Surg*. 2021;34(3):82. doi:10.1053/J.SEMVASCSURG.2021.08.002
6. Fox B. Black and Latino Americans Face Greater Mental Health, Economic Challenges from COVID-19 Than White Americans | Commonwealth Fund. *Commonw Fund*. Published online 2020.
7. Artiga S, Garfield R, Orgera K. Communities of Color at Higher Risk for Health and Economic Challenges due to COVID-19 | KFF. Published online 2020.

8. Smialek J, Tankersley J. Black Workers, Already Lagging, Face Big Economic Risks - The New York Times. Published online 2020.
9. Panchal N, Kamal R, Cox C, Garfield R. The Implications of COVID-19 for Mental Health and Substance Use | KFF. Published online 2021.
10. Riehm KE, Brenneke SG, Adams LB, et al. Association between psychological resilience and changes in mental distress during the COVID-19 pandemic. *J Affect Disord*. 2021;282:381-385. doi:10.1016/J.JAD.2020.12.071
11. Cohen D, Prusak L. *In Good Company. How Social Capital Makes Organizations Work*. Harvard Business School Press; 2001.
12. Saltzman LY, Hansel TC, Bordnick PS. Loneliness, isolation, and social support factors in post-COVID-19 mental health. Published online 2020.
13. Mehta V. The new proxemics: COVID-19, social distancing, and sociable space. *https://doi.org/101080/1357480920201785283*. Published online 2020:669-674. doi:10.1080/13574809.2020.1785283
14. Yang Y, Xiang X. Examine the associations between perceived neighborhood conditions, physical activity, and mental health during the COVID-19 pandemic. *Health Place*. 2021;67:102505. doi:10.1016/J.HEALTHPLACE.2021.102505
15. Losh J, Plyer A. Demographics of New Orleans and early COVID-19 Hot Spots in the U.S. | The Data Center. *Data Cent*. Published online 2020.
16. City of New Orleans. Mayor - News - March 2020 - MAYOR CANTRELL ISSUES STAY HOME MANDATE IN RESPONSE TO COVID-19 - City of New Orleans. Published online 2020.
17. Kessler RC, Andrews G, Colpe LJ, et al. Short screening scales to monitor population prevalences and trends in non-specific psychological distress. *Psychol Med*. 2002;32(6):959-976. doi:10.1017/S0033291702006074
18. Cohen S, Kamarck T, Mermelstein R. A global measure of perceived stress. *J Health Soc Behav*. 1983;24(4):385-396. doi:10.2307/2136404
19. Carman KG, Chandra A, Miller C, et al. Development of the Robert Wood Johnson Foundation National Survey of Health Attitudes : description and top-line summary data. Published online 2016.
20. Sampson RJ, Groves WB. Community Structure and Crime: Testing Social-Disorganization Theory. *https://doi.org/101086/229068*. 2015;94(4):774-802. doi:10.1086/229068
21. De Silva MJ, McKenzie K, Harpham T, A Huttly SR. Social capital and mental illness: a systematic review. *J Epidemiol Community Health*. 2005;59:619-627. doi:10.1136/jech.2004.029678
22. Kobayashi T, Suzuki E, Noguchi M, Kawachi I, Takao S. Community-Level Social Capital and Psychological Distress among the Elderly in Japan: A Population-Based Study. *PLOS ONE*. 2015;10(11):e0142629. doi:10.1371/JOURNAL.PONE.0142629
23. Lebenbaum M, Laporte A, de Oliveira C. The effect of mental health on social capital: An instrumental variable analysis. *Soc Sci Med*. 2021;272:113693.

doi:10.1016/J.SOCSCIMED.2021.113693

24. Umberson D, Karas Montez J. Social Relationships and Health: A Flashpoint for Health Policy. *J Health Soc Behav*. 2010;51(1_suppl):S54-S66. doi:10.1177/0022146510383501
25. Bamford J, Klabbers G, Curran E, Rosato M, Leavey G. Social Capital and Mental Health Among Black and Minority Ethnic Groups in the UK. *J Immigr Minor Health*. 2020;23(3):502-510. doi:10.1007/S10903-020-01043-0/TABLES/5
26. Pitas N, Ehmer C. Social Capital in the Response to COVID-19: *https://doi.org/101177/0890117120924531*. 2020;34(8):942-944. doi:10.1177/0890117120924531
27. Pfefferbaum B, North CS. Mental Health and the Covid-19 Pandemic. *https://doi.org/101056/NEJMp2008017*. 2020;383(6):510-512. doi:10.1056/NEJMP2008017
28. Makridis CA, Wu C. How social capital helps communities weather the COVID-19 pandemic. *PLOS ONE*. 2021;16(1):e0245135. doi:10.1371/JOURNAL.PONE.0245135
29. Harris LK. New Orleans thought Katrina was its worst nightmare - then COVID hit | Reuters. *2020*. Published online 24 8.
30. Childers C, Shaw E, Barsi Z, Mefferd E. Building a Better Future for Women in New Orleans Post COVID-19: Opportunities for Women in Skilled Trade and Technical Jobs - IWPR. *Inst Womens Policy Res*. Published online 2021.
31. Chatlani S. Gulf South health care systems are failing to equally serve people of color, study says | WWNO. *WWNO- New Orleans Public Radio*. Published online 2021.
32. Wakefield SEL, Poland B. Family, friend or foe? Critical reflections on the relevance and role of social capital in health promotion and community development. *Soc Sci Med*. 2005;60(12):2819-2832. doi:10.1016/J.SOCSCIMED.2004.11.012
33. Greenbaum SD. A Comparison of African American and Euro-American Mutual Aid Societies in 19th Century America. *J Ethn Stud Fall*. 1991;19:95.
34. Domínguez DG, García D, Martínez DA, Hernandez-Arriaga B. Leveraging the power of mutual aid, coalitions, leadership, and advocacy during COVID-19. *Am Psychol*. 2020;75(7):909-918. doi:10.1037/AMP0000693
35. Lochner K, Kawachi I, Kennedy BP. Social capital: a guide to its measurement. *Health Place*. 1999;5(4):259-270. doi:10.1016/S1353-8292(99)00016-7
